# Supplementary material for: Adaptive behavior is guided by integrated representations of controlled and non-controlled information
Source: eLife. 2026 Aug 26;14:RP108673. doi: 10.7554/eLife.108673 (PMC13516694; doi:10.7554/eLife.108673)
Supplement: Peer review history

# Editor report: eLife Assessment

**DOI:** 10.7554/eLife.108673.5.sa0

**Authors:** Qing Yu

**Affiliations:** Center for Excellence in Brain Science and Intelligence Technology, Chinese Academy of Sciences China

**Keywords:** Solid, Useful

This **useful** study uses creative scalp EEG decoding methods to demonstrate that two forms of learned associations in a Stroop task are dissociable, despite sharing similar temporal dynamics. The evidence supporting the conclusions is **solid**, although it could be further strengthened with a modified experimental design. This paper would be of interest to researchers studying cognitive control and adaptive behavior.

# Referee report: Reviewer #2 (Public review)

**DOI:** 10.7554/eLife.108673.5.sa1

Summary:

In this EEG study, Huang et al. investigated the relative contribution of two accounts to the process of conflict control, namely the stimulus–control association (SC), which refers to the phenomenon that the ratio of congruent vs. incongruent trials affects the overall control demands, and the stimulus–response association (SR), stating that the frequency of stimulus-response pairings can also impact the level of control. The authors extended the Stroop task with novel manipulation of item congruencies across blocks in order to test whether both types of information are encoded and related to behaviour. Using decoding and RSA they showed that the SC and SR representations were concurrently present in voltage signals and they also positively co-varied. In addition, the variability in both of their strengths was predictive of reaction time. In general, the experiment has an innovative design and the analytical choices are appropriate and the evidence supporting the conclusions are overall solid after taking consideration the control analyses the authors have provided, although within limits of their study design.

Strength:

(1) The authors used an interesting task design that extended the classic Stroop paradigm and is effective in teasing apart the relative contribution of the two different accounts regarding item-specific proportion congruency effect.

(2) Linking strength of RSA scores with behavioural measure is critical to demonstrating the functional significance of the task representations in question.

Comments on revised version.

I appreciate the tireless effort the authors have presented to provide extra control analyses, however, on the other hand I do wish to remind them that sometimes limitations of one study is better addressed by a new study with improved design. There is very good reason why orthogonalization is critical to separating confounding influencing factors which the current study did not completely achieve. Reviewer 1's suggestion on alternative designs is certainly worth considering, and I encourage the authors to continue working on perfecting the experimental design in future work.

# Author comment: Author response

**DOI:** 10.7554/eLife.108673.5.sa2

**Authors:** Bingfang Huang, Harrison Ritz, Jiefeng Jiang

**Affiliations:** University of Iowa Iowa United States; Princeton University Princeton, NJ United States; University of Iowa Iowa City, IA United States

The following is the authors’ response to the previous reviews

**eLife Assessment**

This useful study uses creative scalp EEG decoding methods to attempt to demonstrate that two forms of learned associations in a Stroop task are dissociable, despite sharing similar temporal dynamics. However, the evidence supporting the conclusions is incomplete due to concerns with the experimental design and methodology. This paper would be of interest to researchers studying cognitive control and adaptive behavior, if the concerns raised in the reviews can be addressed satisfactorily.

We thank the editors and the reviewers for their positive assessment and constructive feedback on our work. We also thank the editor for communicating with reviewer #1 regarding our thoughts on their comments. We hence revised the manuscript based the new feedback from reviewer #1. Please see below our responses to each comment raised in the reviews.

**Public Reviews:**

**Reviewer #1 (Public review):**

Summary:

This study focuses on characterizing the EEG correlates of item-specific proportion congruency effects. In particular, two types of learned associations are studied. One association involves associations between stimulus features and control states (SC), and the other involves stimulus features and responses (SR). Decoding methods are used to identify time-resolved SC and SR correlates.

The authors conclude that SC and SR associations can independently and simultaneously guide behavior. This conclusion is based on results showing that SC and SR correlates are (1) not entirely overlapping in cross-decoding, (2) simultaneously observed on average over trials, (3) independently correlate with RT, and (4) have a positive within-trial correlation.

Strengths:

Fearless, creative use of EEG decoding to test tricky hypotheses regarding latent associations.

Nice idea to orthogonalize ISPC condition (MC/MI) from stimulus features.

Thank you for acknowledging the strength in EEG decoding and design. We have addressed all your concerns raised below point by point.

In my view, the ability to address this issue with additional analyses is relatively limited. I cannot think of a solid way to escape this issue in the present design. Adding a nuisance regressor to their RSA regression, which I suggested in my previous response, may reduce the bias, but the efficacy of this would be limited to controlling only a certain kind of phase-dependent confound (a 'main effect' component of study phase, i.e., one that is constant across other conditions; see next message for discussion).

Rather than new analyses, I think a more straightforward revision might be to modify the conclusions advanced in the paper, so that they are more solidly supported by the design and evidence. In my opinion, this design is ill-posed to solidly identify SC and SR representations. As a result, I think that any framing that alleviates pressure on this design to yield 'solid' evidence for identification of SC and SR representations, and instead emphasizes stronger areas of this work, would be constructive.

For example, one potential framing is to advance the idea of SC and SR representations, and discuss an idealized design that could identify them by orthogonalizing stimulus features from ISPC, which are genuinely novel and useful ideas. The current design could then be presented as an opportunistic or initial case study of testing this question, while acknowledging its limitation upfront. The goal here would be to frame the study in a way that allows for the results to be presented with an appropriate grain of salt, while also illustrating the authors thoughtfulness and creativity in devising analyses to test for latent associative representations. In this case the 'solid' label would reference the authors' reasoning and analyses rather than design and conclusions.

I only intend this example as an illustration; there may be several ways of framing this paper so that it is on more 'solid' ground, and I don't want to dictate how exactly authors should write their paper.

Nonetheless I think this issue is important, not only to avoid faulty inference, but also to avoid establishing counterproductive precedents in this field. For example, if students read a paper whose conclusions are labeled "solid" but that nevertheless has critical flaws in its design, then those students may be misled in their own work. But if the flaws were discussed transparently and critically, and the strength of the conclusions were de-emphasized relative to other aspects of the paper, students may not only be inspired by the ideas developed in the paper, but also come away with knowledge about the issues of experimental design.

Discussion of the weaknesses in the conclusions and an additional potential analysis:

The key goal of this study is to identify SC/SR representations, which requires decoupling stimulus features from item-specific proportion congruency (ISPC), but the study-phase confound contaminates this decoupling. I think this impacts both their cross-phase decoding and RSA analyses.

In their results (lines 139-144):

"This standard ISPC manipulation can test whether neural representations of controlled and non-controlled information are wrapped on the same trial by combing with the following EEG analysis (See Methods). However, it potentially mixes color identity with SC, word identity with SR, and the ISPC between SC and SR. To deconfound these factors when estimating SC and SR association representations on each trial, we modified this paradigm by flipping the ISPC contingencies across different phases of the task."

SC/SR representations are higher-order conjunctive classes, formed by the interaction of lower order variables (Color, Word, and ISPC). This means that successfully identifying these representations relies on demonstrating that each class can be reliably individuated from every other class in a manner that cannot be explained by (1) representation of shared lower-order features, such as stimulus color or response, and (2) trivial nuisance factors such as study phase. However, in this design, the trivial factor of study phase is strongly confounded with the ISPC contingency flip.

Regarding RSA: If study phase indeed leads to trivial separability, it seems that similarity among conditions within the same phase would be inflated because the study phase does not appear in the RSA model (Figure 12). In which case, the SC/SR coefficients would be inflated, as the SC and SR models are entirely within-phase.

Nevertheless, an additional control analysis may be possible here. Looking at this regressor set, it seems possible to me to fit a model where the predominant phase is entered as an additional covariate. (If I am reading this correctly, phase would appear as a 2x2 block-diagonal matrix). I suggested this in my last review, but in their recent letter, authors refused. I do not understand why, as I think this regressor set should be identifiable, but perhaps I am wrong here.

That being said, I do not think adding this nuisance covariate would fully solve the issue. The lower-order regressors (Color, Word, ISPC) are defined as the EEG responses shared across phases of the study. To interpret the higher-order SR/SC coefficients, the lower-order components must be fully partialled out. But the putative impact of study phase contaminates this interpretation, as study phase could trivially decrease the similarity of lower-order terms (e.g., decreasing Color similarity between phase 2 vs 3). In which case, the partialling would be expected to be incomplete.

This incomplete partialling is the RSA analogue of the issue in interpreting cross-phase decoding discussed above. As there, so too here I do not see a solid way around it in the present design. This is why in my previous review I referred to the addition of a phase covariate in the RSA regression as a "band-aid": it controls for some problems (main effect of phase), but not all (interactions of phase and lower-order terms).

To summarize, I think that RSA would offer an additional opportunity to control for this potential confound, albeit in a limited sense (study phase effects that are consistent across conditions). But in a more general and rigorous sense, to me, the SC and SR terms in the RSA regression also seem susceptible to the same weakness as the cross-phase decoding analysis.

We thank the reviewer for taking the additional time and effort to provide the new comments. Following the reviewer’s suggestion, we revised the language regarding the conclusions of this project (page 2,5,27-28) and explicitly discussed the weaknesses of the design on decoding and outline a possible solution for future studies in the Discussion section:

“A limitation of the current design is that in theory temporally structured noise (e.g., autocorrelation in EEG data) may bias the decoding accuracy due to the blocked design. Although the present data provided no evidence that the decoding results in this study were biased by temporally structured noise, future studies should aim to develop experimental designs that eliminate this potential confound at the source. One potential solution would be to introduce additional phases flipping ISPC manipulations. At the same time, enough trials must be included in each phase to ensure the strength of the ISPC effect within each phase. A careful balance between session number and length will be helpful to optimize the duration of such a design.”

As eLife also publishes review report, below we also summarize the three control analyses we ran and our reasoning of how they (would) address the issue of temporally structured noise for interested readers to assess:

We acknowledge the theoretical issue of temporally structured noise (TSN) in our design when classes were decoded across different phases. However, the key question for the current data is whether there is empirical evidence that the decoding results were actually driven by TSN. To clarify this issue, we summarize several lines of evidence suggesting that the decoding results were not attributable to TSN:

(1) Split-half cross-validation. We split the EEG data from Phase 2 and the combined Phases 1 and 3 into chronological first and second halves. Phases 1 and 3 were combined because they shared the same MC and MI assignments. This resulted in four possible combinations, each consisting of eight classes drawn from different phases: combination 1 included the first half of Phase 2 and the first half of Phase 3; combination 2 included the first half of Phase 2 and the second half of Phase 3; combination 3 included the second half of Phase 2 and the first half of Phase 3; and combination 4 included the second half of Phase 2 and the second half of Phase 3. We trained the decoders on one combination and tested them on another and then averaged the decoding results across all possible training-test assignments. The similar decoding patterns observed across these analyses (Fig. 6a,b) further confirmed that the decoding results were not driven by TSN.

This analysis is conceptually similar to the “cross-phase” decoding analysis suggested by the reviewer in the first round of review. We also performed an additional distance-based control analysis (see below) to further test whether the decoding results could be explained by TSN, without imposing the constraint used in the split-half cross-validation that trials from the two phases had to fall within a 400-trial window.

(2) Distance analysis. We predicted that if a test trial is closer to a training trial of the same trial type, the higher similarity in TSN between the training and test data would more strongly inflate the decoding accuracy of the test trial, resulting in a negative correlation between distance between a test trial and its closest training trial of the same type and the test trial’s decoding accuracy. However, we did not observe such a negative pattern (Fig. 6c). Note that this distance was defined with respect to trials of the same type, rather than absolute chronological time.

(3) Shuffled analysis. If the decoding results were primarily driven by TSN, either at a short-term or long-term timescale, then shuffling the condition labels within each mini block should preserve the TSN structure present in the real data. In that case, the decoding results from shuffled data should not differ from those observed from real data. However, we found the significant difference between real data and shuffled data as shown in Author response images.

**Author response image 1.** Shuffling analyses with stimulus-locked data support separable SC and SR subspace. (a) Group average decoding accuracy of all 16 experimental conditions as a function of time after stimulus onset. Squares below the lines indicate the significant time points between real data and shuffled data (cluster-based permutation test, cluster-forming threshold p < 0.001, cluster-level p < 0.05). (b) Group average *t* values of representational strength for each factor over time. Squares below the lines indicate the significant time points between real data and shuffled data (cluster-based permutation test, cluster-forming threshold p < 0.001, cluster-level p < 0.05). (c) SC and SR association results from Fig. 1b.

**Author response image 2.** Shuffling analyses with response-locked data support separable SC and SR subspace. (a) Group average decoding accuracy of all 16 experimental conditions as a function of time after stimulus onset. Squares below the lines indicate the significant time points between real data and shuffled data (cluster-based permutation test, cluster-forming threshold p < 0.001, cluster-level p < 0.05). (b) Group average t values of representational strength for each factor over time. Squares below the lines indicate the significant time points between real data and shuffled data (cluster-based permutation test, cluster-forming threshold p < 0.001, cluster-level p < 0.05). (c) SC and SR association results from Fig. 2b.

We thank the reviewer for the suggestion on RSA with phase. There are some concerns for this analysis:

First, we think that the suggested analysis may be difficult to interpret. Because the SC and SR conditions differ across phases. Regressing out phase in RSA could also remove SC and SR information.

Second, based on the reviewer’s comment, we understand that the suggested analysis may still not provide a clear falsifiable criterion for determining whether the results could be driven by the theoretical TSN issue inherent in the design.

Third, the three control analyses we have performed examine this issue from different perspectives and collectively provide no evidence that the results were driven by TSN.

Other readers may, like me, be puzzled by the selection of this particular experimental design to test this question of SC and SR coding, given the temporal confound among SC/SR classes, and given that there would seem to be many possible designs that are less confounded. For example, why not use a design where ISPC was swapped/shuffled several more times within each subject, so that PHASE is more orthogonal to long-timescale noise? Isn't ISPC learning fast enough to support learning phases shorter than 700 trials? Such readers would likely appreciate a frank discussion of this dilemma, and a motivation for the choice of the present design, within the manuscript.

Thank you for your suggestion regarding the design. It is possible that the (re-)learning of ISPC can be fast. That said, enough trials are required to obtain a robust ISPC effect for each phase after the ISPC flips. Given that the EEG scanning (not including capping) in current design was about 1.5 hours, it is impractical to have both more sessions for a more orthogonal design and long sessions for robust within-session ISPC effects. We chose to maximize the latter because flipped behavioral ISPC effect in each session is the basis for the following EEG analysis. We have included the reviewer’s suggestion as a potential design solution for future studies in the Discussion section mentioned above on page 27.

Pre-stimulus coding:

To explain the apparent pre-stimulus coding of several task variables, the newest version of the manuscript proposes that subjects were proactively coding these variables via predictive mechanisms. This is an interesting account of item-specific control. It is also surprising, given that item-specific control mechanisms are typically conceptualized as reactive or stimulus-driven phenomena. But I think support for a proactive control account was incomplete. The mechanistic logic was not presented, and no hypotheses under this account were developed or tested. So I would suggest pinning down some hypotheses here and actually putting this account to the test.

Thank you for raising this important point. Although ISPC effects are considered reactive, in our design the long sessions may create a temporal context for the participants to differentiate the current control demand linked to each color. The maintenance of such contextual information needs to span across trials, leading to pre-stimulus coding that proactively guides the control demand for each color. This claim is not central to this manuscript, which investigates whether SC and SR representations simultaneously guide behavior. Additionally, we do not think the current design is well-equipped to test this hypothesis because the pre-stimulus onset is the only supporting evidence. In the revised manuscript, we discussed this as a future research direction and proposed a design that aims at better isolating proactive control signal on page 25.

Random slopes were omitted due to convergence failure, but this can inflate false positive inferences (e.g., Barr et al. 2013), and doesn't really motivate a minimal model. I'd suggest trying a slightly reduced model (e.g., drop correlations via `slope || subject`) using buildMer automated selection, or switching to brms.

We indeed tried both the full model of random effects (i.e., considering covariance between all slopes and intercept) and a reduced model without any covariance (i.e., listing each random slope separately without intercept in lme4). However, neither model converged at all time points.

**Reviewer #2 (Public review):**

Summary:

In this EEG study, Huang et al. investigated the relative contribution of two accounts to the process of conflict control, namely the stimulus–control association (SC), which refers to the phenomenon that the ratio of congruent vs. incongruent trials affects the overall control demands, and the stimulus–response association (SR), stating that the frequency of stimulus-response pairings can also impact the level of control. The authors extended the Stroop task with novel manipulation of item congruencies across blocks in order to test whether both types of information are encoded and related to behaviour. Using decoding and RSA they showed that the SC and SR representations were concurrently present in voltage signals and they also positively co-varied. In addition, the variability in both of their strengths was predictive of reaction time. In general, the experiment has a solid design and the analyses are appropriate for the research questions.

Strengths:

(1) The authors used an interesting task design that extended the classic Stroop paradigm and is effective in teasing apart the relative contribution of the two different accounts regarding item-specific proportion congruency effect.

(2) Linking the strength of RSA scores with behavioural measure is critical to demonstrating the functional significance of the task representations in question.

We thank you for acknowledging our work on design and brain-behavior analysis. We have addressed your concerns raised below.

Weaknesses:

I still have some doubts on the effectiveness of the experimental manipulation on Phase 2: although the ISPC effect is still present, it is much weaker in comparison, suggesting the participants did not learn the contingency statistics in Phase 2 as well as they did in the other phases, due to either the lingering effect of the previous phase or an inherent bias towards one color pairs. Perhaps by separately plotting the earlier and later blocks of Phase 2 any difference can be revealed if it exists. This behavioral difference could result in unequal levels of SC/SR representation across phases, which may raise problems when data were combined for analyses that assume the neural effects are equivalent.

Thank you for your concern about this important issue. We agree with the reviewer that the true SR/SC levels may not be equivalent between Phase 2 and Phase 1/3. Nevertheless, because the manipulation of ISPC is binary, the decoders were trained to test whether the neural signals represent the two levels of ISPC (i.e., a higher vs. a lower level) differ systematically. The decoding analysis does not require that the neural effects of SC and SR must be numerically equivalent between phases (i.e., it is not necessary that the two levels are equidistant from the center point of SC/SR. Indeed, the decoding analysis only requires that the two levels are different). For example, if the ISPC level ranges from -1 to 1 and the EEG signals can reliably decode ISPC levels of -0.5 and 0.7 (i.e., two unequal levels), it can still be treated as supporting evidence that ISPC levels are encoded in the EEG signals. The same logic applies to the representational subspace analysis. As to the RSA, as can be seen in Fig. 12, the regressors are also binary, encoding whether two experimental conditions share the same SC/SR level without assuming equivalent neural effects. Thus, we argue that the reported decoding and RSA can still test the encoding of SR and SC. We discussed this issue on page 24.

Following the reviewer’s comment, we plotted the ISPC effects in first and second half of Phase 2 separately (the figure below). We also tested whether the ISPC effects differ qualitatively between the two halves using a 3-way ANOVAs separately on RT and Error rate. The results showed that the time (the first half vs. the second half) × Congruency × ISPC interaction was not significant for either RT data (*F*_(1,39)_ = 3.40, *p* > 0.05) or error rate (*F*_(1,39)_ = 2.49, *p* > 0.05), suggesting that ISPC effect did not systematically change over time in Phase 2 See Supplementary Figure 9.
